# Medical Opinion and Movement

**Published:** 1909-12-04

**Authors:** 


### Medical Opinion and Movement.

JAUNDICE of the new born has formed the subject of research by Dr. C. Hasse, and an account of his observations appears in the *Jahrbuch für Kinderheilkunde*. Many are the theories that have been conceived to account for the origin of this transient and apparently innocuous jaundice of the new-born infant, one of the most generally accepted being that it is hæmolytic in origin, and due to the decomposition of excess of blood received from the maternal organism just before division of the cord. Dr. Hasse is of opinion, however, that it is a normal biliary jaundice due to a temporary stasis of the bile. This stasis of the bile he explains on anatomical lines after a careful study of the condition of the liver and the adjoining parts at the time of birth of the infant. In fœtal life, and for a little while after birth, the liver is of considerable volume, and predominates over the abdominal organs. From his careful study of the liver at this time the author draws particular attention to the condition of its inferior surface, and especially the quadrate lobe and the lobe of Speighel. About half of this corresponds to the inferior surface of the organ, but the Speighelian lobe comes so far forward that the transverse fissure is curved backwards, and as it were buried. This extension forwards pushes the upper segment of the duodenum forwards under the quadrate lobe, and makes it take a direction from before backwards and from above downwards under the portal fissure. From these anatomical relations it follows that the bile duct, the hepatic artery, and the portal vein take a course in the form of an S, rising first horizontally forwards, then downwards, and finally backwards, enclosed between the quadrate lobe, the Spieghelian lobe, the pancreas, and the first part of the duodenum. The respiratory movements of the new-born infant, causing depression of the diaphragm at each inspiration, raise the blood-pressure in the portal vein and so produce compression of the bile ducts; and at the same time the liver is compressed against the anterior abdominal wall and against the subjacent viscera. From this compression there result biliary stasis and jaundice from absorption. But as normal respiration becomes established the circulation of the blood through the liver is facilitated, the congestion of the liver is relieved, the organ conforms more to its normal condition, the pressure on the bile ducts is relieved, and the bile flows into the intestine freely and without obstruction.

---

DR. R. KLAPP, of Berlin, has devised a new method of operation for mobilising an Ankylosed Knee-joint. The method depends upon the fact that in ankyloses of this joint the posterior aspect of the femoral joint surface is usually uninvolved. The principle of operation consists in the utilisation of this area for a new joint surface. An account of the operation and of two cases successfully treated by the method appears in the *Zentralblatt für Chirurgie*. The adhesions are first broken down under anæsthesia to the point of extreme flexion. With the knee-joint held at right angles a vertical incision is made on the lower and inner aspect of the femur and a large wedge is removed from the bone. The wedge removed is so made that its base is anterior and includes the upper part of the anterior joint surface of the bone, while the angle lies just above the upper limit of the posterior joint surface. When this wedge is removed and the sawn surfaces of the bone are approximated, the articular end of the femur is rotated forwards and upwards, so that the posterior articular surface now becomes inferior. In bringing together the sawn surfaces care must be taken that the tibial aspect of the femoral surface is not disturbed from its position. The first patient treated in this way is able to flex his knee-joint to the right-angled position now, six months after the operation. The second operation, which from an operative point of view was a perfect success, has been performed too recently to warrant any definite conclusions.

---

ONE of the latest therapeutic purposes to which hydrogen peroxide is applied consists in the treatment of Gastric Hyperchloridia. Dr. Petri, of Halle, first drew attention to the effect of hydrogen peroxide on gastric secretion. More recently Dr. Goodman, of Philadelphia, has pursued the subject by giving the test meal of Ewald, in which the usual cup of tea was replaced by 50 cubic centimetres of a 0.5 per cent. solution of peroxide of hydrogen. His observations were carried out on fifteen patients, and in all of them he found that the hydrogen peroxide had a marked inhibitory effect upon the secretion of hydrochloric acid. In six patients there was a total absence of free hydrochloric acid, without causing the patients any inconvenience. Following these observations Dr. Goodman has tried the effect of this treatment in cases of hypersecretion of hydrochloric acid. He gives about half an hour after meals a large glass of water, with one or two teaspoonfuls of a 3 per cent. solution of hydrogen peroxide. He has treated in this way four patients who showed all the classic symptoms of hyperchloridia, and in every case the treatment resulted in diminishing the pains and acid eructations and in reducing the amount of free hydrochloric acid. In one case the results appeared particularly remarkable, as every other known remedy had been previously tried without success. Dr. Goodman is therefore of opinion that we have in hydrogen peroxide a valuable medicament against the distressing symptoms of gastric hypersecretion of hydrochloric acid.

---

AT a meeting of the Académie des Sciences Dr. A. Monvoisin gave the results of his research work on the Milk of Tuberculous Cows. He has analysed samples of milk from seven tuberculous cows, some of which had tuberculous mammæ, others had well-marked visceral lesions, but were without any mammary lesion that could be detected by the most careful clinical examination. He finds that the milk supplied from a tuberculous udder passes insensibly in composition from the normal to that of blood serum, both in regard to organic and inorganic constituents. When the affection is well advanced the mammary epithelium allows to pass whatever is brought to it by the blood without modifying it. Thus whereas normal cow's milk contains in 1,000 cubic centimetres 38.5 parts of proteid, 46.5 of fat, 43.5 of sugar, 1.4 of sodium chloride, and 7.3 of other inorganic salts, the milk from tuberculous cows contains 72.4 of proteid, 0.7 of fat, 0 to 2 of sugar, 5.1 of sodium chloride, and 9.6 of other inorganic salts.

---

The ingenuity of trans-Atlantic obstetricians and gynæcologists, and their fertility of invention have often been illustrated in these pages by the description of novel methods and ideas from their continent. The latest new suggestion is as bold, but certainly not so practicable, as any that have preceded it; for it is nothing less than an endeavour to anticipate and prevent Dystocia by operative enlargement of the contracted pelvis (Pubiotomy) in the non-pregnant state. Dr. J. N. Bell, of Detroit, advanced the project before the recent meeting of the American Association of Obstetricians and Gynæcologists, and the arguments with which he tentatively proposed it are mainly four. First, when pubiotomy is done during labour, or at term, the turgid condition of the pelvic vessels causes risk of grave hæmorrhage. Next, by operating in the non-pregnant state more care can be exercised to secure a clean field of operation, and the danger of sepsis is thus diminished. Again, injury to the vaginal attachments and to the bladder due to the passage of the child's head against the severed ends of the bone, and undue separation of the divided ends are all avoided. Lastly, the difficulties of handling the patient after the operation at term are materially lessened. The indications given are: a history of two or more difficult deliveries with the child born dead, unsuccessful results of induction of premature labour, a conjugate of 7.5 to 8.5 c.m., a refusal of Cæsarean section, and a strong desire for a living child. The operation has been done once only, upon a woman who had never been pregnant, but was curetted in the hope she might become so. At the same time pubiotomy was done because of contraction of the pelvis, and an increase of 1 cm. in the transverse diameters of the pelvis was secured. The operation was condemned by all those who joined in the subsequent debate.

---

A discussion of the precise moment at which a Fulminating case of Ruptured Tubal Gestation should be submitted to laparotomy is reported in the *American Journal of Obstetrics and Gynæcology*. These cases comprise what obstetricians are prone to allude to as "the five per cent." That is, the statistics of widely separated clinics all over the world show that in very nearly this proportion the first symptoms of rupture are those of a most alarming and dangerous hæmorrhage. It follows that the patients when seen are usually in a state of profound shock, and the exact routine most suited to them and most likely to be of benefit has been for some time a matter of contention. On one hand are those who urge that the correct surgical treatment of grave hæmorrhage is the immediate aseptic ligature of the bleeding vessels: that means, in this lesion, immediate laparotomy. On the other those who argue that even in the worst cases it is most unusual for the patient actually to bleed to death of the primary hæmorrhage; that operation during severe collapse has greater dangers than conservatism for a little while; and that the best thing to do is to secure absolute rest, to administer small doses of morphia, and to remove the gestation sac twenty-four or forty-eight hours later with the patient in a much better general condition, and with the superior facilities for aseptic surgery which a few hours' delay and preparation render possible. This view has been maintained both upon clinical experience in practice, and by arguments based upon experiments with animals. In the latter it has been shown that it is almost impossible to kill a bitch by division of the uterine and ovarian arteries of both sides: the hæmorrhage soon ceases spontaneously, and the animal recovers without any very serious symptoms.

---

These views have been very ably advanced by Robb and others in papers which have on previous occasions been abstracted in these columns (The Hospital, March 20, 1909, p. 632; October 31, 1908, p. 118). They are stoutly opposed by Dr. Huggins, the author of the paper which led to the discussion now under review. This surgeon narrates two recent cases in which he withheld his hand for a few hours in order to avoid operating during the shock of a large primary hæmorrhage, and in which a second hæmorrhage occurred very shortly after. One of the cases ended fatally before anything could be done; the other he was able to save by a hasty laparotomy. He has investigated the coagulability of the blood in fifty dogs of many breeds and both sexes, and he finds it to be just half that of human blood estimated by the same instrument; this, he thinks, may account for the experimental results on which the conservatives base their system. He also repeated Robb's experiments on pregnant bitches, and one of the ten operated upon died, while in others the hæmorrhage was quite severe. Without dealing with the treatment of the 95 per cent. of less grave cases, Huggins is emphatic that for the others instant operation under light ether anæsthesia is the proper treatment. Saline infusion is to be begun during the operation, which should not require more than fifteen or twenty minutes. The extreme Trendelenburg position is to be avoided; and the limbs of the patient are to be firmly bandaged. The surgeons who took part in the debate supported this outline of treatment unanimously, or almost so.

---

The Acetone Treatment of Inoperable Carcinoma is favourably reported upon in the *Medical Record* by Dr. Tovey in a lengthy article which is an interesting contribution to cancer literature. This method was devised by Gellhorn of St.

Louis, who was led to adopt it by the powerful properties of hardening pathological specimens which this substance displays. It is indicated in any inoperable cancer of the uterine cervix or of the surface of the body, when the breaking-down area can be submitted to its direct action. First of all the ulcerating mass is to be thoroughly scraped out under anæsthesia, otherwise the acetone is uselessly spent in hardening dead tissue. The curetted cavity or crater is then carefully dried with gauze sponges, and half an ounce or more of acetone poured in (through a Ferguson's speculum in the case of the cervix, and in the Trendelenburg position). After half an hour the fluid is allowed to escape, and the cavity is then packed with gauze soaked in acetone. Care should be taken not to allow the acetone to run over healthy mucous membrane or skin, since that gives rise to a good deal of after-pain and discomfort; if owing to the circumstances of the case it is difficult to prevent this, a preliminary anointing with vaseline is desirable. The immediate effect of the application is the cessation of all bleeding: the surface of the crater is covered with a white film. There is great diminution in the discharge, and a marked reduction in its fœtid odour. The general condition of the patient improves quickly as a rule, but pain due to extension of the disease into adjacent organs or nerves is not relieved. Acetone cannot be found in the urine of those thus treated. After the first sitting there is no need to repeat the scraping, but the rest of the programme is repeated twice a week.

---

It would seem likely that Vaccination and Preventive Immunisation of the patient is the surgical procedure of the future, and it is quite within the bounds of possibility that the time is not far distant when the surgeon will be able, prior to operation, to immunise his patient against all risks of infection. Some recent experiments of Dr. Th. Tuffier to this end have been reported in *La Presse Médicale*. This observer remarks that, although it is not possible at the moment to place one's patient in a condition of immunity, a greater degree of resistance to infection can be induced by suitable treatment. When infection has taken place there develops in the individual attacked a hyperleucocytosis. The function of these newly-arrived white cells is to kill the invading micro-organisms and to destroy or neutralise their toxins. If, therefore, it were possible before operation to induce a considerable leucocytosis, infection would be almost impossible. For general leucoprophylaxis 20 c.cm. of a 1 per cent. solution of nucleinate of soda, or horse-serum, heated to 55° C. are injected subcutaneously. In some cases an intravenous injection of 5 c.cm. of a four-volume solution of tallianine is made. To bring about a local reaction, a half-per-cent. solution of nucleic acid or ordinary physiological serum is injected.

The author has found that no marked results have followed these therapeutic measures. Local leucoprophylaxis is difficult of application, especially in abdominal surgery, for the necessary puncture is not free from danger, and a double operation with a short interval between is by no means desirable. The value of general leucoprophylaxis has not yet been proved, although it is harmless in application. In grave conditions it fails to attain its object, and in the less serious states it is not certain whether the hyperleucocytosis thus provoked is of the same defensive value as that brought about by the infective agent itself. Moreover, the efficiency of the leucocytosis does not depend so much on the number as on the combative value of the polynuclear leucocytes, and it is not proved that the concourse is an army at all.

---

Kermisson of Paris has recently had the opportunity of examining two cases of Facial Coloboma. In the first there existed a simple coloboma, consisting of an oblique slit which started from the upper lip and ended in a furrow dividing the lower eyelid of the same side into two equal parts. In the second case the deformity was bilateral. Two oblique slits starting from the upper lip joined the lower eyelid as in the first case, thereby isolating from the rest of the face the mass corresponding to the two nares. The author insists on the very probable influence which amniotic adhesions may have on the pathogenesis of these deformities. The existence in the second case of a fairly deep congenital furrow just above the right wrist is a further point in favour of this hypothesis.

---

It is well enough known that the ordinary Mist. Ferri Co. of the British Pharmacopœia changes colour within a few days of its being made up, owing to oxidation of the ferrous sulphate. One method of preventing this is to add a considerable excess of sugar to the mixture—oxidation of the sugar being, so to speak, a relief to the ferrous sulphate. It may, however, be undesirable to give sugar in this way, either because the patient does not like it, or because it is contra-indicated on account of diabetes mellitus. A simple method of prescribing the mixture in two portions which, kept separately, remain good, and which, mixed together in much the same way as one mixes Fehling's Solutions Nos. 1 and 2, together form fresh Mist. Ferri Co., has lately been described by Mr. T. D. Sneath in a letter to the *Pharmaceutical Journal*. The ordinary directions are that the myrrh, potassium carbonate, and sugar are used with 7 oz. of rose water, and the ferrous sulphate with 3 oz. of rose water. Mr. Sneath makes a solution using double quantities of the myrrh, potassium carbonate, and sugar, and 15 oz. of rose water, putting the result into an amber-coloured glass-stoppered bottle labelled "Mist. No. 1, pro Mist. Ferri Co. B.P." Next he uses 5 oz. of rose water instead of 6, the double quantity required, dissolving the ferrous sulphate in the 5 oz. of rose water and putting this product into another amber-coloured glass-stoppered bottle labelled "Mist. No. 2, pro Mist. Ferri Co. B.P." One part of solution No. 2 with three parts of solution No. 1 forms Mist. Ferri Co. B.P.

